# Functional Outcome of a Coronal Shear Fracture of the Capitellum Managed by Herbert Screw Fixation Using the Anterolateral Surgical Approach

**DOI:** 10.7759/cureus.6578

**Published:** 2020-01-06

**Authors:** Sitender Garg, Arnab Sain, Vijay Sharma, Kamran Farooque, Namith Rangaswamy

**Affiliations:** 1 Orthopaedics, All India Institute of Medical Sciences, New Delhi, IND

**Keywords:** coronal shear fracture, capitellum, anterolateral approach, open reduction, internal fixation, herbert screw

## Abstract

Fractures of the capitellum, particularly coronal shear fractures, are difficult to manage. The challenges are adequate surgical exposure, proper anatomic reduction, and stable fixation of these fractures. Our study included 10 patients with a coronal shear fracture of the capitellum without any involvement of the posterior condyle. All patients underwent open reduction and Herbert screw fixation using the anterolateral approach, with good functional outcome. In our opinion, this is a good option for the treatment of coronal shear fractures of the capitellum.

## Introduction

Capitellar fractures are extremely rare injuries. They constitute about 1% of elbow fractures [1]. The mechanism of injury is usually axial loading through the radial head. Both conservative and operative methods have been tried for the management of these fractures [2]. In the present scenario, open reduction and internal fixation, which provides a stable congruent joint, is the most preferred treatment strategy. However, because of the complexity of these intra-articular fractures, surgical exposure and fracture fixation are difficult [2-9].

In a coronal shear capitellar fractures, the lateral approach is commonly used for exposure of the elbow [2-9]. But with the lateral approach, the exposure of the medial articular extension and trochlea is inadequate. So it is difficult to delineate the actual anatomy of the fracture. The anterolateral approach of the elbow joint has been adapted in many studies to treat these capitellar fractures. The anterolateral surgical approach provides adequate anterior exposure of the elbow joint as compared to the lateral approach [10-11]. 

Regarding the fixation of the capitellar fracture, various implants, such as mini-fragment screws, Herbert screws, and bioabsorbable rods, have been used. Herbert screw fixation provides compression at the fracture site, stable fixation, and no intra-articular prominence of the implant [2,12-20]. There is no accepted guideline regarding the method of internal fixation for these fractures [2].

In our study, we used the anterolateral approach to assess the functional outcomes in 10 cases with coronal shear capitellar fractures fixed by Herbert screws. Our aim is also to investigate the outcome and safety of this surgical procedure. Our hypothesis was that open reduction and internal fixation of the coronal shear capitellar fractures with Herbert screws through the anterolateral approach of the elbow is an excellent method of treatment for these fractures.

## Materials and methods

**Figure 1:**
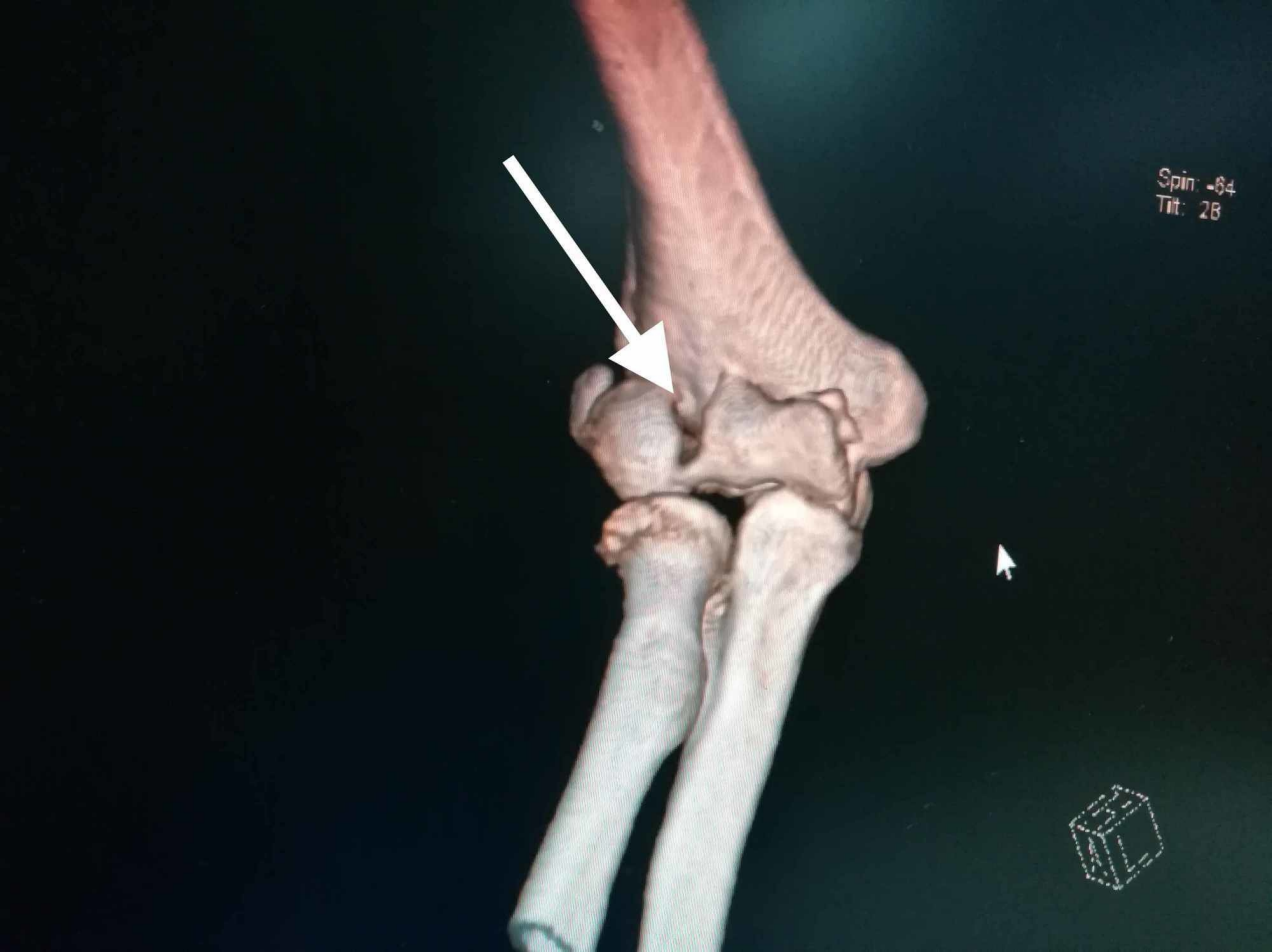
Computed tomography (CT) scans with a 3D reconstruction helps in the better assessment of the fracture profile

Our study included 10 consecutive adult patients from June 2017 to July 2018 with coronal shear capitellar fractures. None of the patients had the involvement of the posterior condyle along with a fracture of the capitellum. Preoperative consent was taken for all patients and approval from the department was taken. All the patients were followed up for a minimum of 12 months. They were all treated with open reduction and internal fixation with Herbert screws by the anterolateral surgical approach within five days of trauma. Open fractures were excluded from our study. The mechanism of injury, clinical examination, and radiographic data were recorded in each patient. Preoperatively, anteroposterior (AP) and lateral X-ray radiographs of the elbow were taken. Computed tomography (CT) with 3D reconstruction was done for a better assessment of the fracture profile and to rule out other injuries (Figure 1). The Dubberley classification system [5] was used to classify the fractures (Table 1).

**Table 1:** Dubberley classification system

Type of Fracture	Fracture anatomy
Type 1	Fracture which involves only the capitellum +/- involvement of the trochlear ridge
Type 2	Fracture which involves the capitellum and the trochlea as a single fragment
Type 3	Fracture which involves both the capitellum and the trochlea as separate fragments

Surgical technique

Anesthesia and Position

All the patients in our study underwent regional anesthesia or general anesthesia depending on the preference of the anesthesia team. Patients were positioned supine with the involved upper extremity over the broad radiolucent armrest and a tourniquet was tied around the proximal aspect of the arm. We checked for elbow ligamentous instability in all patients under anesthesia in the operating room just before the start of surgery.

Surgical Approach

**Figure 2:**
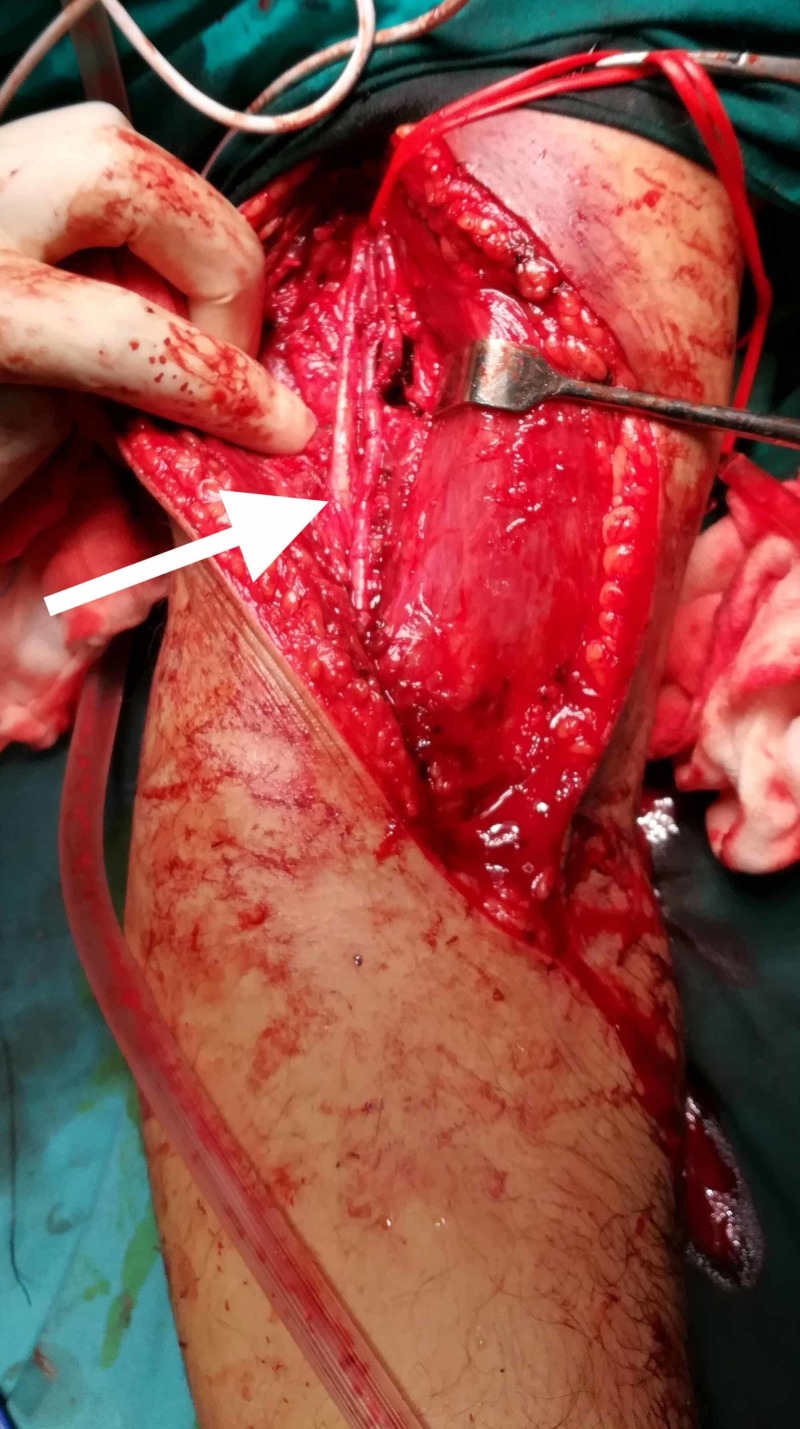
Anterolateral approach for exposure of the elbow joint. Please note the important neurovascular structures indicated by the arrow

The surgical approach was adopted as described in the study by previous studies [13,18,20]. The incision started 3-4 cm proximally above the elbow crease extending following the lateral border of the biceps muscle. The incision was then extended medially across the elbow joint obliquely and then extended in the proximal forearm following the medial border of brachioradialis for about 8 cm. We avoided crossing the elbow flexion crease at 90 degrees. The surgical dissection continued between the biceps and brachioradialis origin by blunt dissection. In the superficial plane, care was taken to identify the antebrachial lateral cutaneous nerve and protect it. Then, the radial nerve was visualized on the deep surface of the brachioradialis and carefully retracted laterally to protect it throughout the duration of surgery. The biceps muscle and brachialis muscle was retracted medially and the anterior capsule of the elbow joint was exposed. The capsule was cut longitudinally for fracture site exposure (Figure 2).

Fracture Reduction and Fixation

**Figure 3:**
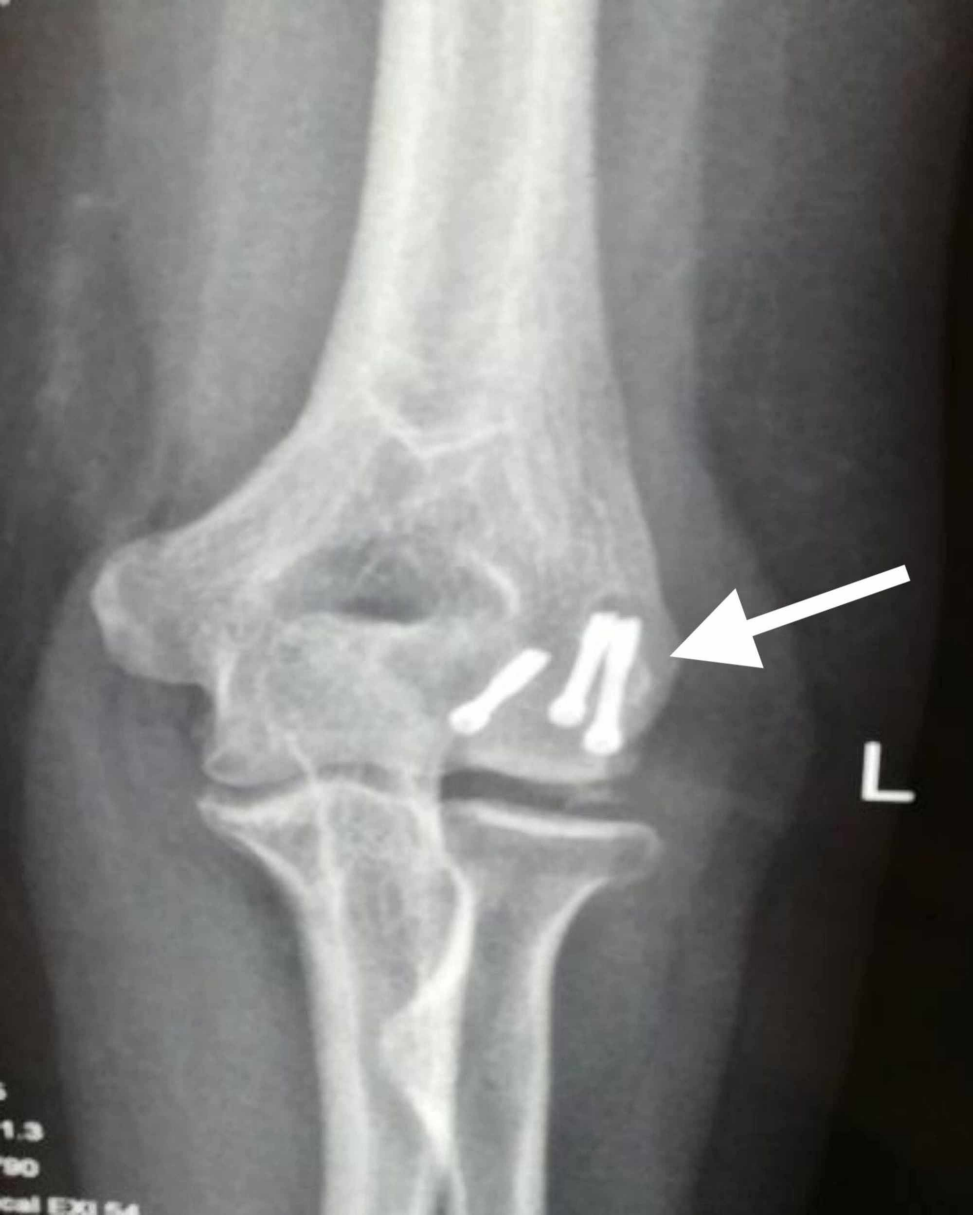
Post-operative X-ray radiograph anteroposterior(AP) view showing a fracture of the capitellum managed with Herbert screw fixation

**Figure 4:**
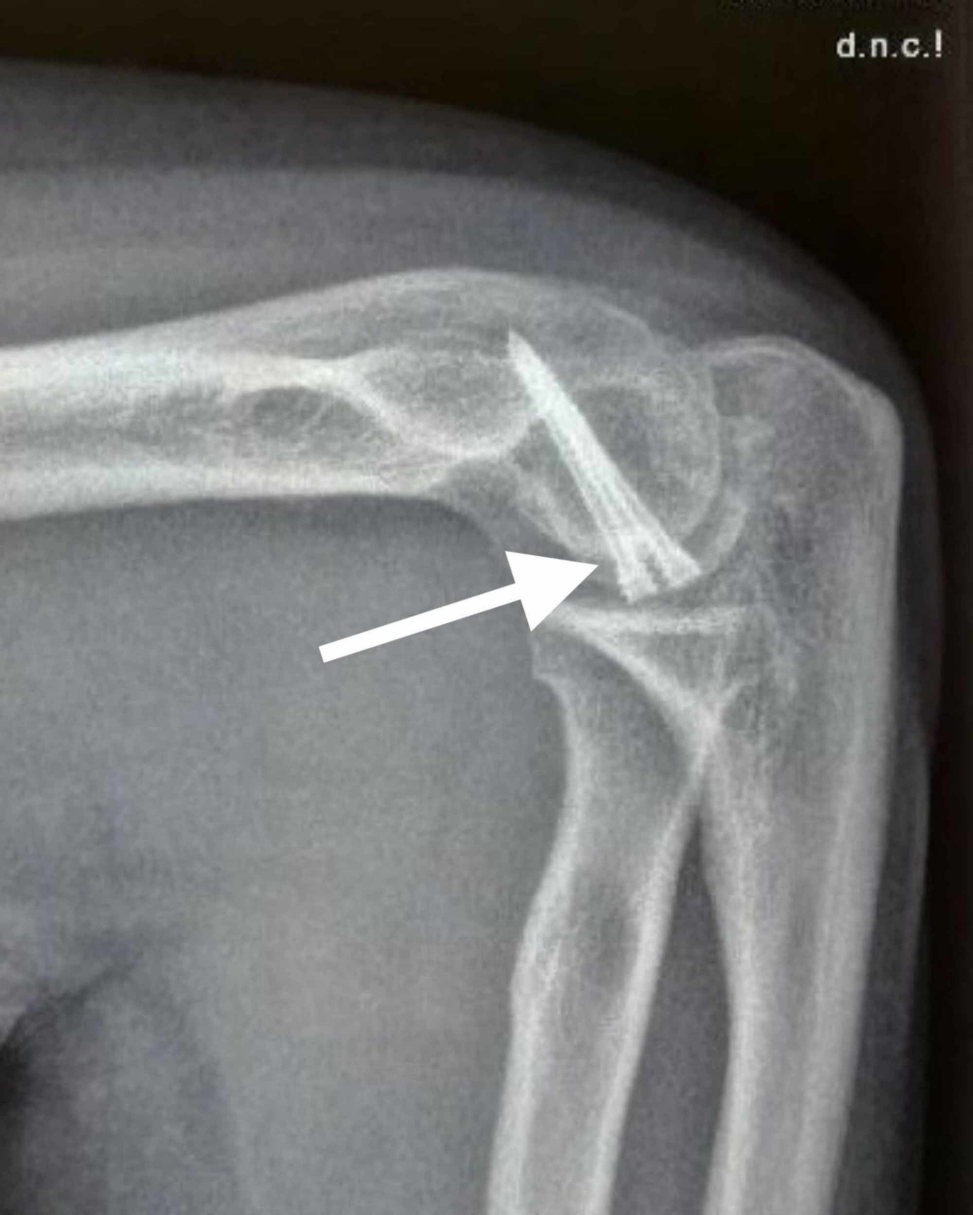
Postoperative X-ray radiograph lateral view showing a fracture of the capitellum managed with Herbert screw fixation

After exposure of the fracture site, thorough irrigation was done to remove the blood clots. Sometimes, for better visualization of the medial and lateral extent of the fracture, a Hohmann retractor was placed along the supracondylar ridge of the distal humerus. The anatomical reduction of the fracture was then done by matching the articular fracture lines and stabilized provisionally with multiple guidewires. The reduction was then checked with fluoroscopy. Then, after drilling over guidewires, Herbert screws were inserted over the guidewires and the screws were buried under the articular cartilage. The elbow was then assessed for the full passive range of motion and stability. After the final assessment of the fracture reduction and position of screws with fluoroscopy, wound closure was done in layers and a negative pressure suction drain was used. Below are the images showing X-ray radiographs of capitellar fractures managed with Herbert screw fixation (Figures 3-4).

Post-operative Care

The elbow was immobilized in an above-elbow posterior splint to provide relief from postoperative pain. The elbow was kept at 90° of flexion and neutral rotation for five days in the splint. Adequate postoperative analgesia was given after consultation with the anesthesia team. The drain was removed after 48 hours and the active range of motion (ROM) of the elbow was started after five days.

Follow-Up

Serial follow-up was done postoperatively at approximately one, three, six, and 12 months and patients were assessed clinically and radiographically. During every follow-up, the elbow range of motion, pain, and stability of the elbow joint were assessed clinically. Also, Mayo Elbow Performance Index (MEPI) scoring [2] was done at 12 months for every patient. Serial radiographs were done to evaluate the healing of the fracture, post-traumatic osteoarthritis, and avascular necrosis. Here is a video demonstrating the postoperative ROM of the elbow joint in an operated case of capitellar fracture managed with Herbert screw fixation (Video 1).

**Video 1:** Video demonstrating the postoperative range of motion of the elbow joint in an operated case of capitellar fracture managed with Herbert screw fixation through the anterolateral approach at 12 months follow-up

## Results

Eight patients were male, and two were female. The mean age was 29.3 years (range, 21 to 42 years). Four patients developed fractures due to falls, and six patients had a fracture of the capitellum due to road traffic accidents. There was no incidence of problems with wound-healing problems or infection. All fractures healed well in the anatomic position as confirmed on radiographs. During the final follow-up, two patients had mild pain. None of the patients had any complaints about the instability of the elbow. The flexion-extension range of motion was 136° ± 10° in the affected elbow and 145° ± 5° in unaffected elbow respectively. The supination-pronation range of motion was 173° ± 11° in the affected elbow and 178° ± 3°in the unaffected elbow, respectively. The values suggest that the ROM between the affected and unaffected elbows did not differ much. The average MEPI Score was 96 ± 4 (range 90 to 100). All 10 patients were satisfied and were able to return to their pre-injury activity levels. None of the patients had complications like post-traumatic arthritis of the elbow joint or avascular necrosis of the capitellum. One patient had developed a posterior interosseous nerve injury that recovered fully after six months. Below is the table showing patient demographics, mode of injury, range of motion, and MEPI score at follow-up (Table 2).

**Table 2:** Patient demographics, mode of injury, range of motion, MEPI Score at follow-up MEPI Score: Mayo Elbow Performance Index (MEPI) Score; RTA: road traffic accident

Patient	Age	Gender	Mechanism	Follow-up months	Range of motion (flexion/extension in degree)	Range of motion (supination/pronation in degree)	MEPI score
1	24	M	Fall	16	140	170	100
2	30	M	RTA	14	135	180	95
3	42	F	Fall	14	140	165	100
4	22	M	RTA	12	130	170	95
5	28	M	RTA	14	135	180	100
6	34	M	Fall	16	125	180	95
7	38	M	RTA	12	140	170	90
8	29	F	RTA	14	145	165	95
9	21	M	Fall	12	135	180	90
10	25	M	RTA	14	140	170	100

## Discussion

Fracture type and complexity and the preference of the operating surgeon form the basis of the selection of surgical approaches. The aim of preserving the blood supply of the fracture fragment also takes part in operative approach selection [2]. The lateral approach of the elbow and the anterolateral approach of the elbow joint are the preferred approaches in coronal shear capitellar fractures. The most commonly used surgical approach for exposure of the elbow as per previous studies is the lateral approach. In this approach, exposure to the elbow joint is done by elevation of the common extensor origin from the lateral epicondyle [2]. However, the disadvantage of the lateral approach is a lack of sufficient exposure of the capitulum and trochlea, and this hinders the visibility of the fracture site for anatomic reduction. Also, inserting the screws perpendicular to the fracture site by this approach is difficult (particularly for fractures of the capitellum extending to the trochlea) [2,10-11,13]. Dubberley et al. recommended that sectioning of the lateral collateral ligament or a flexor-pronator split is needed if the medial aspect of the trochlea cannot be exposed adequately or if the anatomic reduction is not ensured by the lateral approach [5]. The anterolateral approach has the advantage that it can expose the capitellum and trochlea clearly, which makes it possible to reduce the fracture anatomically and achieve stable fixation by screw placement perpendicular to the fracture line [2,10-11,13]. Besides, the release of the common extensor origin is avoided, preventing postoperative extensor lag. So the use of the anterolateral approach of the elbow can prevent the pitfalls of the lateral approach of the elbow [2].

In our experience, the anterolateral approach provides sufficient exposure of the elbow joint anatomy, especially the joint cartilage of the medial aspect of the elbow joint. By this approach, it is possible to achieve the anatomic reduction of the fracture with screws placed in the anterior to posterior direction. The only drawback of this anterolateral approach of the elbow is that the plane of dissection is very close to the vital anatomic neurovascular structures in the elbow, and it carries a risk of injury to the radial nerve during surgery. Most studies on the anterolateral approach reported no injury of the neurovascular structures. However, Vaishya et al. reported one incidence of posterior interosseous nerve palsy in the postoperative period, which recovered completely [13]. In our opinion, the radial nerve should be exposed in the anterolateral approach of the elbow and then retracted carefully and protected throughout the duration of surgery. Direct visualization of the nerve followed by careful retraction reduces the chance of radial nerve injury.

The implant used for fracture fixation is also an important area of interest. Various implants like the metallic lag screws, headless screws, bioabsorbable rods, dorsal plates, and fibrin glue were previously used for the reconstruction of capitellar fractures. Herbert screw fixation is now the most commonly preferred technique. Studies on joint biomechanics have demonstrated that Herbert screws can give good stability of fixation for capitellum fractures. Although several different types of screws like the cannulated, headless, cortical, and cancellous screws have been used, no direct comparison between fixation with different types of screws are available. This is due to the heterogeneous reporting of clinical outcomes with different implants. The advantages offered by Herbert screws are adequate fracture compression, stable anatomic fixation, and no intra-articular prominence of the implant [2,13,15-20]. In our study, the MEPI Score had good to excellent results (96 +/- 4), and it was similar to those previously published studies [2,9,14]. Although two patients reported mild pain during activity, all patients had a satisfactory functional outcome and they were able to return to pre-injury activity levels.

Ligamentous injuries are commonly associated with fractures of the elbow and can lead to elbow instability. Giannicola et al. reported elbow instability in coronal shear capitellar fractures associated with elbow dislocation. This injury pattern was associated with injuries of the lateral collateral ligament (LCL) and medial collateral ligament (MCL). So ligamentous stability must be assessed in these cases [2-3]. The study by Mighell et al. reported LCL injuries with Dubberly type 2A fractures of the capitellum but none in Dubberly type 1 fractures [2,12]. Are et al. also found no LCL or MCL injury in Dubberly type 1 fractures of the capitellum [2,6]. In our study, no associated LCL or MCL injuries were found as checked in the operating room after anesthesia was given to the patient. In our view, ligamentous injuries generally occur in capitellar fractures associated with elbow dislocations. It is recommended to rule out any ligamentous instability by examination under anesthesia.

Also, no incidence of post-traumatic osteoarthritis of the elbow joint and avascular necrosis of the capitellum was reported in our case series, which is similar to several studies [6,18-19]. In our view, if the soft tissues attached to the capitellum is preserved, proper reduction and stable fixation with less damage to the articular cartilage is done, the incidence of these complications is minimal. In our opinion, satisfactory functional outcomes in coronal shear capitellar fractures can be made possible by accurate anatomic reduction, stable fixation, and early start of the ROM exercises. This can be achieved with ease by the use of Herbert screw fixation through the anterolateral approach of the elbow.

The limitation of our study was the small number of patients and the short period of follow-up. A greater number of patients and a follow-up of a longer duration is required to find out the true incidence of post-traumatic arthritis of the elbow joint and avascular necrosis of the capitellum.

## Conclusions

Our findings suggest that open reduction and Herbert screw fixation using the anterolateral approach is an excellent method of treatment for coronal shear capitellar fractures. This method can achieve stable fixation of the fracture and restoration of a functional range of motion of the elbow.
